# Time trends in the use and appropriateness of natriuretic peptide testing in primary care: an observational study

**DOI:** 10.3399/bjgpopen20X101074

**Published:** 2020-08-12

**Authors:** Mark Valk, Arno W Hoes, Arend Mosterd, Brenda Broekhuizen, Nicolaas Zuithoff, Frans H Rutten

**Affiliations:** 1 Julius Center for Health Sciences and Primary Care, University Medical Center Utrecht, Utrecht, The Netherlands; 2 Department of Cardiology, Meander Medical Center, The Netherlands, Amersfoort

**Keywords:** Heart failure, primary care, natriuretic peptides, time trend, diagnosis

## Abstract

**Background:**

Diagnosing heart failure (HF) is difficult, relying on medical history, symptoms, and signs only. Clinical guidelines recommend natriuretic peptides (NPs) as an additional diagnostic test, notably to exclude HF in suspected patients. NP testing has been available since 2003 for primary care in the Netherlands, but little is known about its uptake.

**Aim:**

To evaluate the trend in ordering and appropriateness of NP testing in primary care.

**Design & setting:**

An observational study was performed between January 2005 and December 2013. Nine Dutch general practices participated, with 21 000 registered people (approximately 4300 aged ≥65 years).

**Method:**

The total number of patients undergoing NP testing each year was calculated per 1000 patient years (PY) based on the total practice population. NP levels were used to assess whether NP testing was applied to exclude or confirm HF.

**Results:**

The number of NP testing increased from 2.5 per 1000 PY in 2005 to 14.0 per 1000 PY in 2013, with a peak in 2009 of 15.6 per 1000 PY. The proportion of participants with N-terminal B-type natriuretic peptide (NTproBNP) below 125 pg/ml (the exclusionary threshold recommended by the European Society of Cardiology [ESC] guidelines on HF) was on average 30%, and highest in the first year (47%).

**Conclusion:**

After a rapid uptake of NP testing in primary care from 2005 onwards, the use of it seemed to stabilise after 2009, thus leaving patients who are prone to HF without an optimal diagnostic work-up.

## How this fits in

Diagnosing HF is difficult when only relying on medical history, symptoms, and signs. Guidelines recommend NP as an additional test in suspected patients. The use of NP testing for exclusion of HF diagnosis and opportunistic screening should be considered by GPs more often. After a rapid uptake of NP testing in primary care from 2005 onwards to 2009 it stabilised, although there is room for improvement.

## Introduction

HF is an important health problem, and adequate management starts with a correct diagnosis. However, when relying on medical history, symptoms, and signs only, diagnosing HF is notoriously difficult.^1,2^ Electrocardiography provides relevant information, but is not generally available in general practice, and access to echocardiography is even more limited. In clinical guidelines, NPs, notably B-type (BNP) and amino-terminal B-type (NTproBNP) are recommended for the initial diagnostic assessment of patients suspected of HF immediately following history taking, signs, and symptoms, to exclude HF and select those requiring echocardiography to confirm the diagnosis.^3^

NPs were first identified in the porcine brain in 1988.^4^ Measurement of NP levels in patients became available in hospital laboratories around 2002, and in 2003 Dutch GPs could order this testing in laboratories. In 2005, the Dutch primary care guideline on HF recommended for the first time BNP or NTproBNP testing for the initial diagnostic assessment of patients with symptoms and signs suggestive of HF in the primary care setting.^5^ The updated 2010 Dutch primary care HF guidelines,^6^ and also the 2012 and 2016 ESC guidelines on HF,^3,7^ specifically mentioned the importance of NP testing (and electrocardiography) as a means to exclude the presence of non-acute HF (very high negative predictive value), and, therefore, recommended a very low exclusionary cut-point (NTproBNP <125 pg/ml, BNP <35 pg/ml) below which other diagnoses than HF should be considered, and above which patients should be referred for echocardiography.^3,7^ The National Institute for Health and Care Excellence (NICE) guidelines on HF, however, based their NTproBNP cut-point 400 pg/ml on the best accuracy of this test (optimal balance sensitivity and specificity).^8^ At that cut-point fewer patients will be referred for echocardiography and, in those referred HF, will be detected more often but at the price of missing patients with HF, notably those with preserved ejection fraction with NTproBNP values below 400 pg/ml. Recent studies clearly demonstrated on the one hand that many (40%–80% in some high-risk groups) older patients with HF in primary care have not been recognised as such (underdiagnosis),^9–12^ while on the other hand patients with a GP’s label of HF in 17% did not really have HF, according to an expert panel using all available diagnostic information (overdiagnosis).^13^ Around 30% of patients suspected of HF by the GP and referred to a cardiologist actually have HF based on a full diagnostic work-up (including echocardiography).^2,14^

Currently, there is very limited data on the uptake of NP testing in primary care over time. Against this background, the study investigated the time trend in ordering NP testing by GPs in the Netherlands from 2005 to 2013.

## Method

GPs from nine primary care practices in Soest, a city in the vicinity of Utrecht, the Netherlands, participated in the study between March 2005 and December 2013. In the Netherlands, all inhabitants are registered with a GP, irrespective of cooperative care of a hospital specialist, except for patients living in a nursing home or hospice. In the participating practices, 21 000 individuals were enlisted (approximately 4300 aged ≥65 years). All patient contacts and specialist letters were kept by the GP in an electronic medical record (EMR).

**Figure 1.**
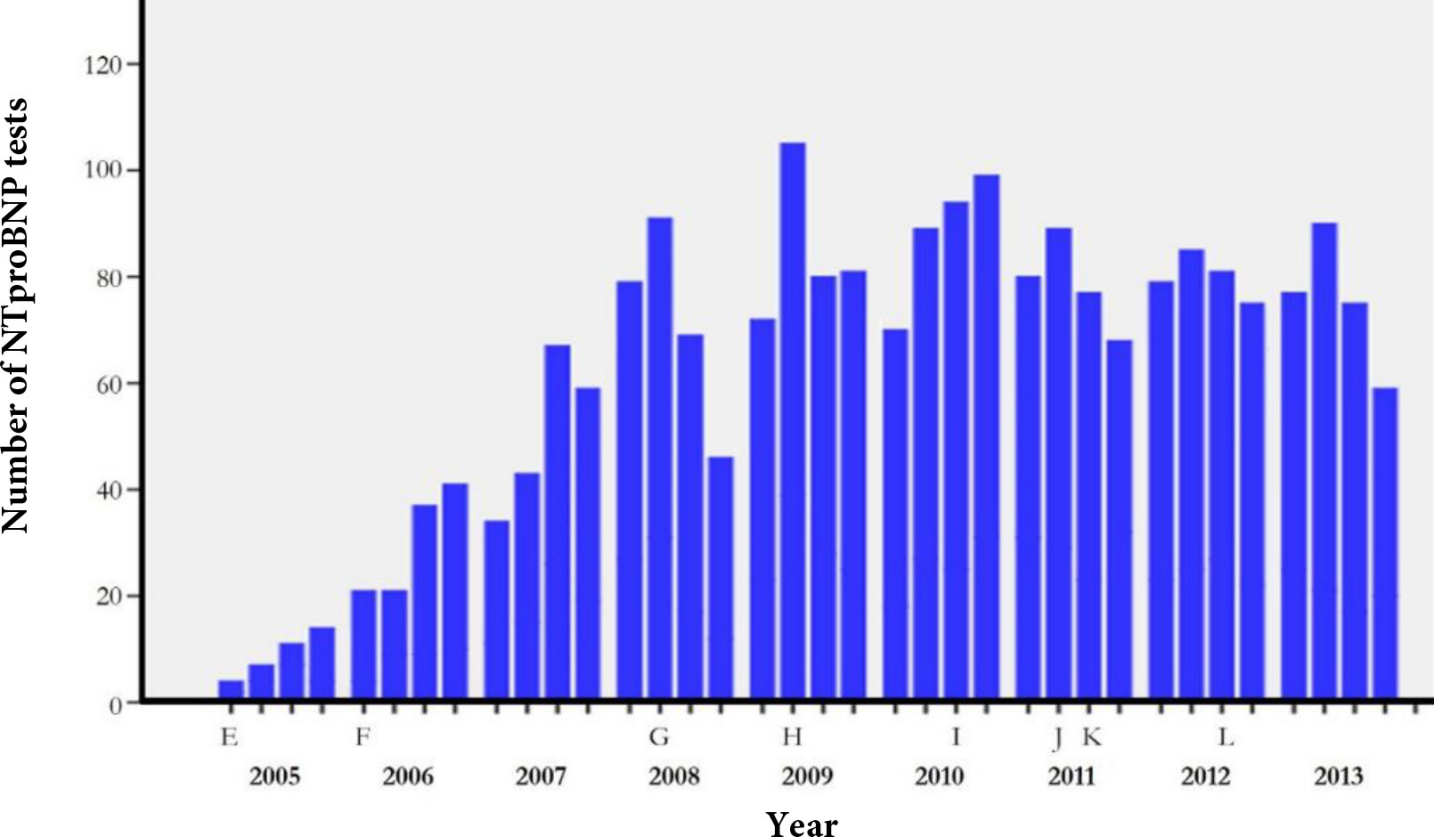
Number of N-terminal B-type natriuretic peptide (NTproBNP) tests as was ordered per quarter of the year by GPs in the period January 2005 until December 2013. The letters E to L correspond to the following events: (E) 2005: Update of the Dutch GPs’ guidelines on heart failure (HF), now mentioning NTproBNP and BNP as an option in the diagnostic assessment. Also in this year an update of the European Society of Cardiology (ESC) guidelines on HF. (F) 2006: Patient reimbursement stopped for laboratory testing. (G) 2008: Update of the ESC guidelines on HF, clearly recommending natriuretic peptides (NPs) for diagnosis, but without advocating a cut-point. (H) 2009: Single-day training on the diagnosis of HF and the use of NTproBNP of the participating GPs of Soest. (I) 2010: Update of the Dutch GPs’ guidelines on HF, now recommending the use of the NTproBNP exclusionary cut-point of 125 pg/ml (approximately 15 mmol/l). If values are below this threshold, and the electrocardiogram is normal, than HF is very unlikely and other diagnoses should be considered to explain the symptoms of patients. (J) 2011: Regional agreement on open-access echocardiography in the Soest area. (K) 2011: Regional agreement between GPs and cardiologists on HF referral, diagnosis, and management in the Soest area. (L) 2012: Update of the ESC guidelines on HF, now also explicitly recommending the exclusionary cut-point of 125 pg/ml (approximately 15 mmol/l) for NTproBNP

All NP measurements ordered by the participating GPs between January 2005 and December 2013 were extracted from the Meander Medical Center hospital database. In this region, NP ordering became available for GPs in 2003, with the hospital laboratory of Meander Medical Center as the regional supplier. This laboratory used NTproBNP, and it was measured on the Elecsys analyser (Roche Diagnostics). Results were given in pg/ml. In Figure 1 events between 2005 and 2013 are summarised that may have affected the ordering of NTproBNP tests.

The total number of enlisted people and the proportion aged ≥65 years were calculated for each year.

The participating GPs agreed to the use of de-identified patient data, and signed informed consent. The study was conducted in accordance with the Law for the Protection of Personal Data and confirmed to the principles outlined in the Declaration of Helsinki.^6,14,15^

### ​Data analysis

The number of NTproBNP tests ordered was calculated per 1000 PY for each year between 2005 and 2013. NTproBNP values were dichotomised at 125 pg/ml (approximately 15 mmol/l) to determine the proportion of tests that likely served to exclude HF.

## Results

During the 9-year period, 2269 NTproBNP measurements were ordered by the participating GPs; in 2005, 2.5 orders per 1000 PY, increasing in subsequent years to 15.6 orders per 1000 PY in 2009, with stabilisation afterwards. At the end of the study period in 2013, 14.0 tests were ordered per 1000 PY (Figure 1).

**Figure 2.**
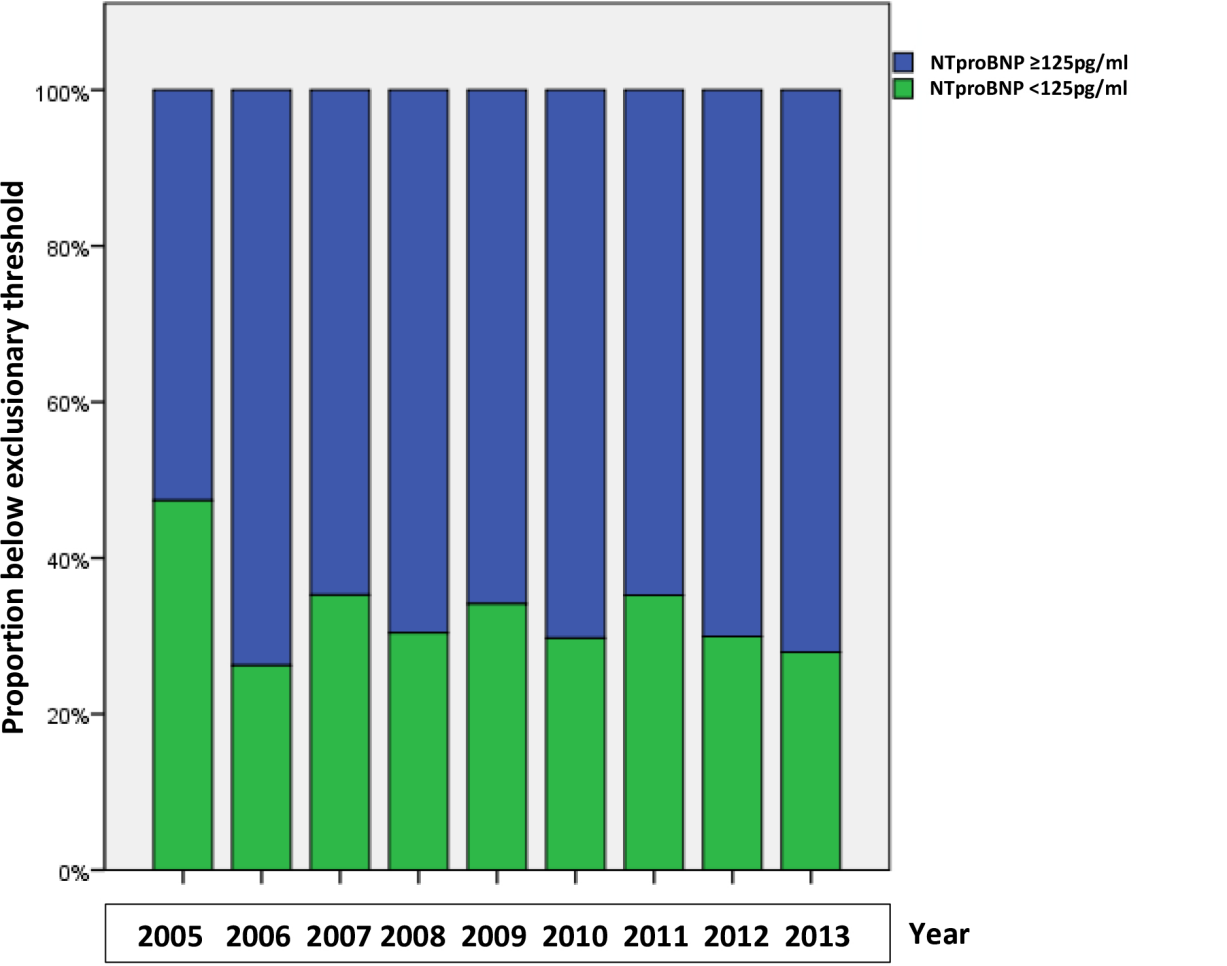
Proportion of the participating GPs ordered NTproBNP values above and below the exclusionary threshold of 125 pg/ml for each year from 2005–2013

The proportion of NTproBNP results below 125 pg/ml was on average 30% during the study period. The highest was in 2005 with 47% followed by a decline in 2006 to 26%, and remaining the following years in the range 28%–35% until the end of the study in 2015 (Figure 2).

## Discussion

### Summary

NP testing by Dutch GPs increased steeply from 2.5 per 1000 PY in 2005 to 15.6 per 1000 PY in 2009, followed by a stabilisation in the subsequent years (14.0 per 1000 PY in 2013). The availability of B-type NPs and guideline recommendations for their use in the diagnostic assessment seems to have contributed to the increase in NP ordering by GPs (see Figure 1). The peak in 2009 may additionally be related to the 1-day training on diagnosing and treating HF, as was provided to the participating GPs in that year.

### Strength and limitations

The strength of the study was that all participating GPs sent their requests to one single hospital laboratory. A limitation was that the individual patient data could not be assessed. Patient characteristics could, therefore, not be provided nor exclusions made when GPs ordered multiple NP measurements over time, in some cases for monitoring. Such monitoring, however, is certainly not common practice in Dutch primary care.

### Comparison with existing literature

To the best of the authors' knowledge, there are no other studies published in international medical journals about time trends in NP testing in primary care. A small study on time trends was published in a Dutch medical journal providing data over the period 2004–2007, also showing an increase in NP testing, from 1.0 per 1000 PY in 2004 to 6.5 in 2007.^16^

In the present study, on average 30% had NTproBNP values below 125 pg/ml, and that is similar to two other studies in primary care patients suspected of HF published in 2005: 42% and 24%.^14,17^ In the latter study, however, only patients with a reduced left ventricular ejection fraction were considered, not those with HF and a preserved ejection fraction (HFpEF). In patients with HFpEF, lower NTproBNP levels can be expected than in those with HF with reduced ejection fraction (HFrEF).^7,14,18^

It is known that a minority (around 30%) of patients who are referred by their GP because of suspected HF based on medical history, symptoms, and signs actually have HF.^2,14^ Conditions other than HF but related to shortness of breath may also result in NTproBNP levels above 125 pg/ml; for example, atrial fibrillation, renal dysfunction, left ventricular hypertrophy, and age >75 years.^3^

Thus, there seems to be room for improvement of the efficiency of NP testing in primary care. In addition, opportunistic screening should be considered in people with an increased risk of HF; for example, older people from the community with type 2 diabetes, chronic obstructive pulmonary disease (COPD), or those who visit the practice for shortness of breath, or have multimorbidity and polypharmacy.^9–12^

### Implications for practice

Indirectly, the data suggest that GPs do not optimally use NP measurements. This readily available test could be considered in anybody suspected of HF, but also as an opportunistic screening tool in those aged >60–65 years with COPD or type two diabetes.

NP testing by Dutch GPs increased steeply from 2005–2009, with a stabilisation from that time onwards. The use of NP testing for exclusion of HF diagnosis and opportunistic screening should be considered more often by GPs.
